# Supplementary material for: The Crosstalk Analysis between mPSCs and Panc1 Cells Identifies CCN1 as a Positive Regulator of Gemcitabine Sensitivity in Pancreatic Cancer Cells
Source: Int J Mol Sci. 2024 Aug 29;25(17):9369. doi: 10.3390/ijms25179369 (PMC11394772; doi:10.3390/ijms25179369)
Supplement: Supplementary file 1 [file ijms-25-09369-s001.zip › ijms-3100724-supplementary.pptx]

## Slide 1
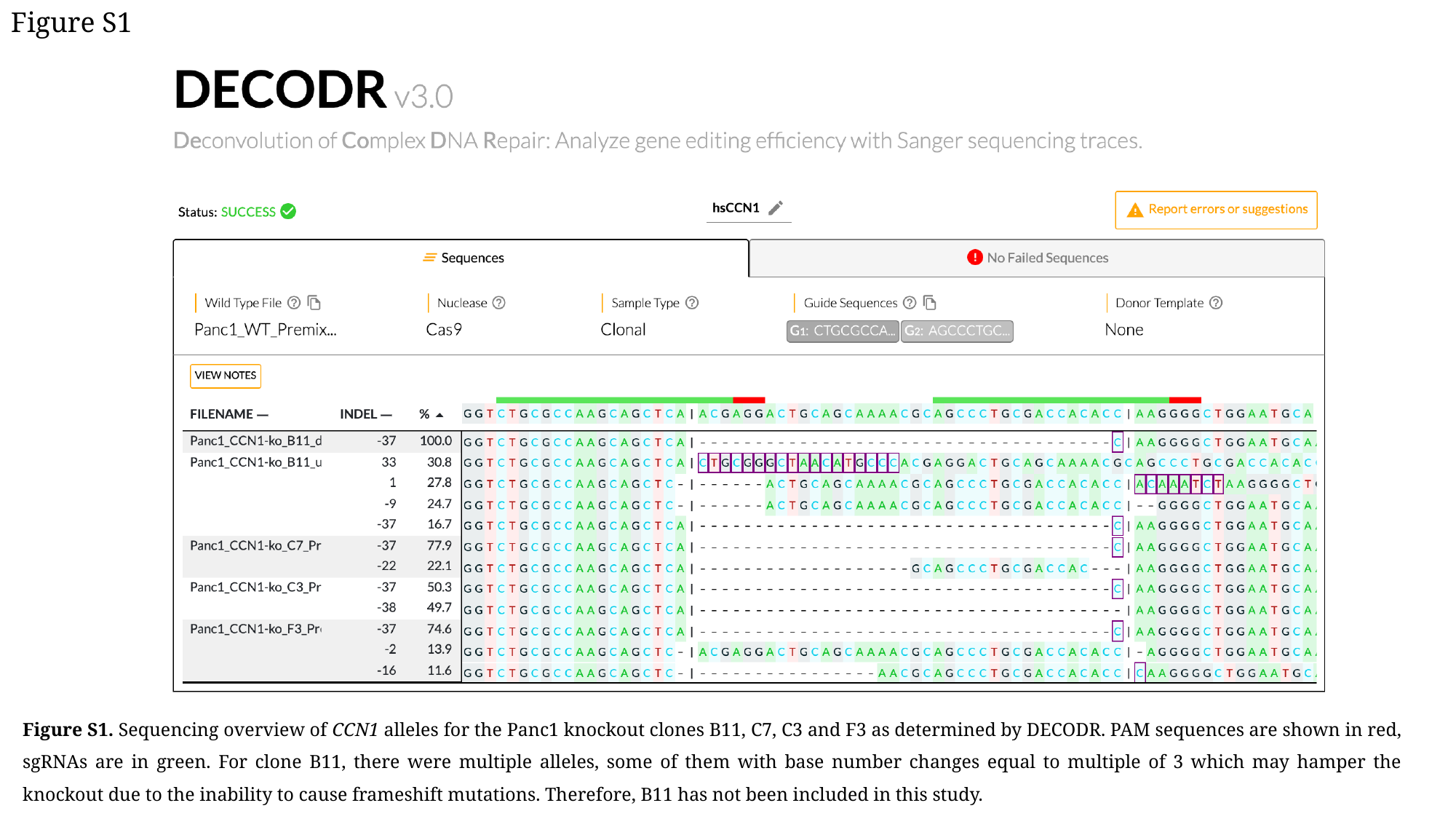

Figure S1
Figure S1. Sequencing overview of CCN1 alleles for the Panc1 knockout clones B11, C7, C3 and F3 as determined by DECODR. PAM sequences are shown in red, sgRNAs are in green. For clone B11, there were multiple alleles, some of them with base number changes equal to multiple of 3 which may hamper the knockout due to the inability to cause frameshift mutations. Therefore, B11 has not been included in this study.

## Slide 2
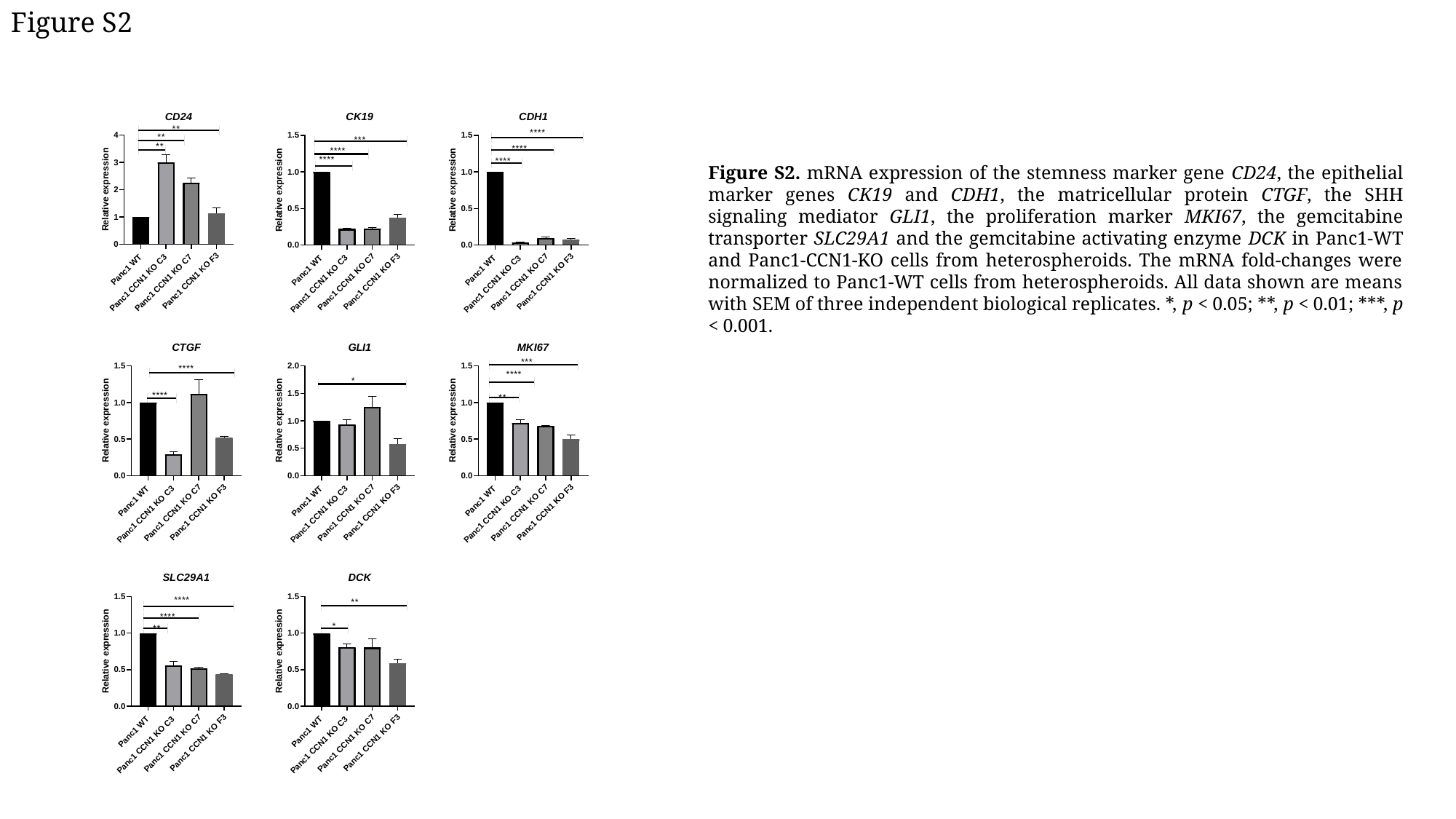

Figure S2
Figure S2. mRNA expression of the stemness marker gene CD24, the epithelial marker genes CK19 and CDH1, the matricellular protein CTGF, the SHH signaling mediator GLI1, the proliferation marker MKI67, the gemcitabine transporter SLC29A1 and the gemcitabine activating enzyme DCK in Panc1-WT and Panc1-CCN1-KO cells from heterospheroids. The mRNA fold-changes were normalized to Panc1-WT cells from heterospheroids. All data shown are means with SEM of three independent biological replicates. *, p < 0.05; **, p < 0.01; ***, p < 0.001.

## Slide 3
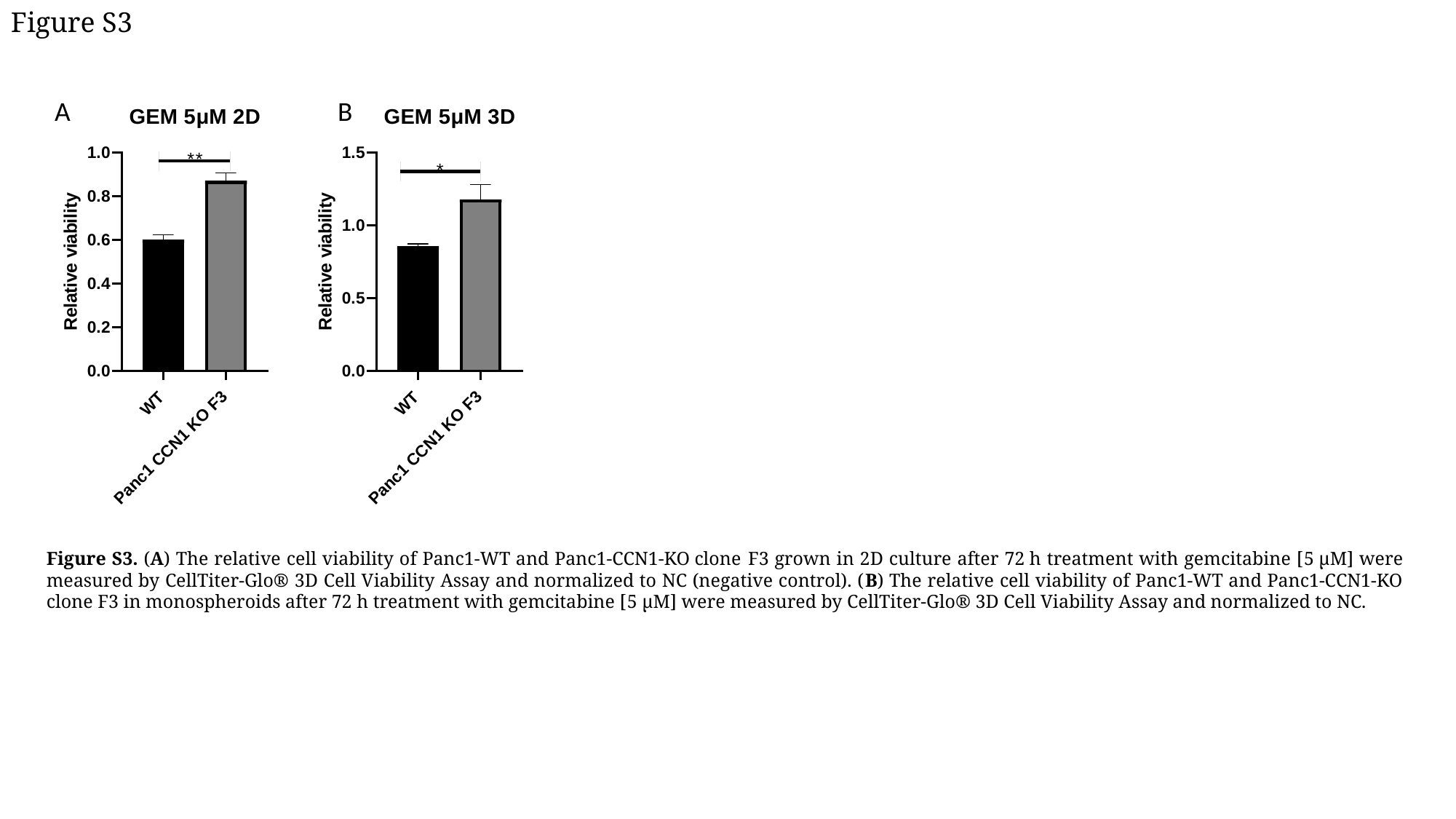

Figure S3
A
B
Figure S3. (A) The relative cell viability of Panc1-WT and Panc1-CCN1-KO clone F3 grown in 2D culture after 72 h treatment with gemcitabine [5 μM] were measured by CellTiter-Glo® 3D Cell Viability Assay and normalized to NC (negative control). (B) The relative cell viability of Panc1-WT and Panc1-CCN1-KO clone F3 in monospheroids after 72 h treatment with gemcitabine [5 μM] were measured by CellTiter-Glo® 3D Cell Viability Assay and normalized to NC.

## Slide 4
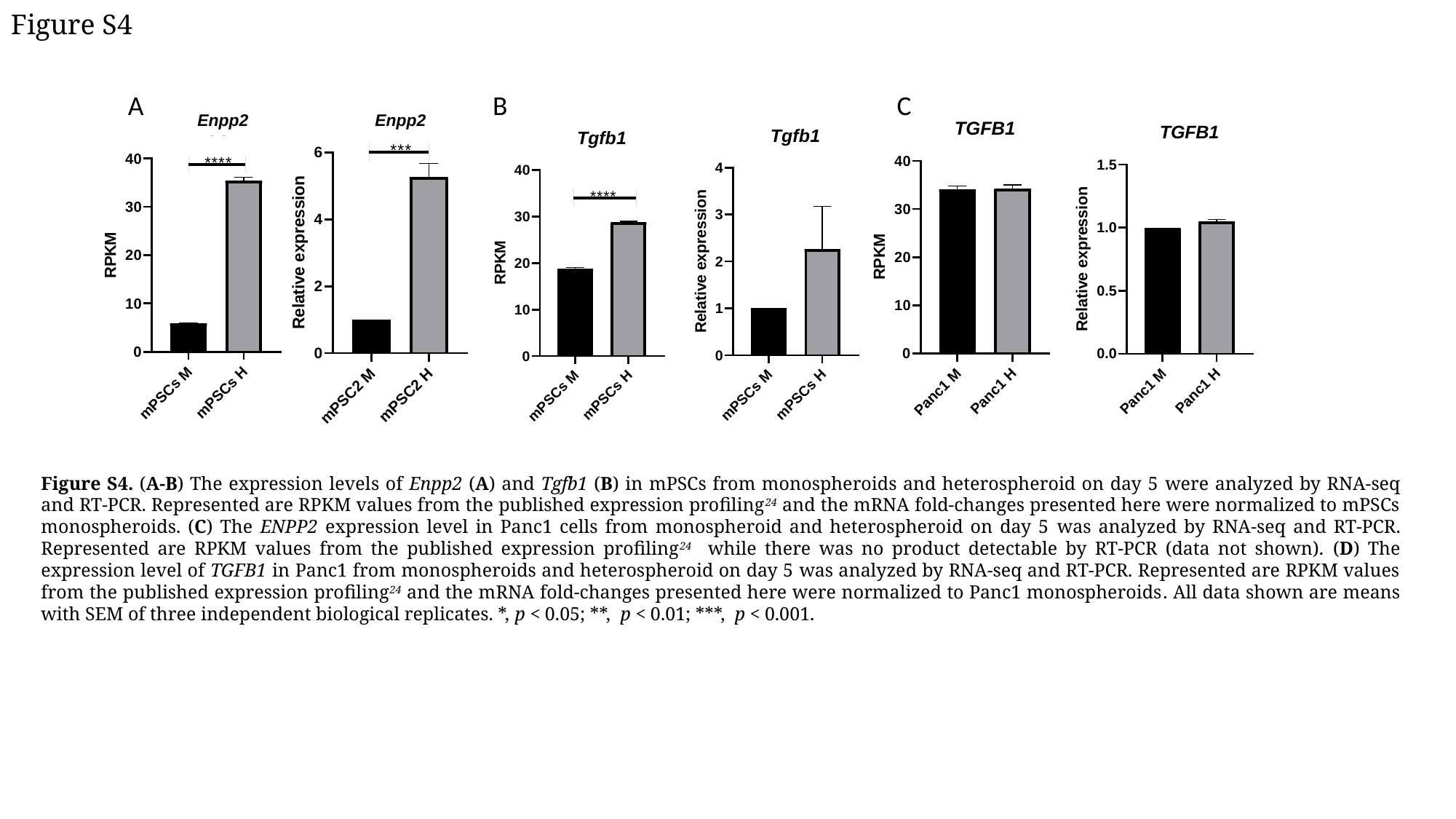

Figure S4
A
B
C
Enpp2
Enpp2
Figure S4. (A-B) The expression levels of Enpp2 (A) and Tgfb1 (B) in mPSCs from monospheroids and heterospheroid on day 5 were analyzed by RNA-seq and RT-PCR. Represented are RPKM values from the published expression profiling24 and the mRNA fold-changes presented here were normalized to mPSCs monospheroids. (C) The ENPP2 expression level in Panc1 cells from monospheroid and heterospheroid on day 5 was analyzed by RNA-seq and RT-PCR. Represented are RPKM values from the published expression profiling24 while there was no product detectable by RT-PCR (data not shown). (D) The expression level of TGFB1 in Panc1 from monospheroids and heterospheroid on day 5 was analyzed by RNA-seq and RT-PCR. Represented are RPKM values from the published expression profiling24 and the mRNA fold-changes presented here were normalized to Panc1 monospheroids. All data shown are means with SEM of three independent biological replicates. *, p < 0.05; **, p < 0.01; ***, p < 0.001.

## Slide 5
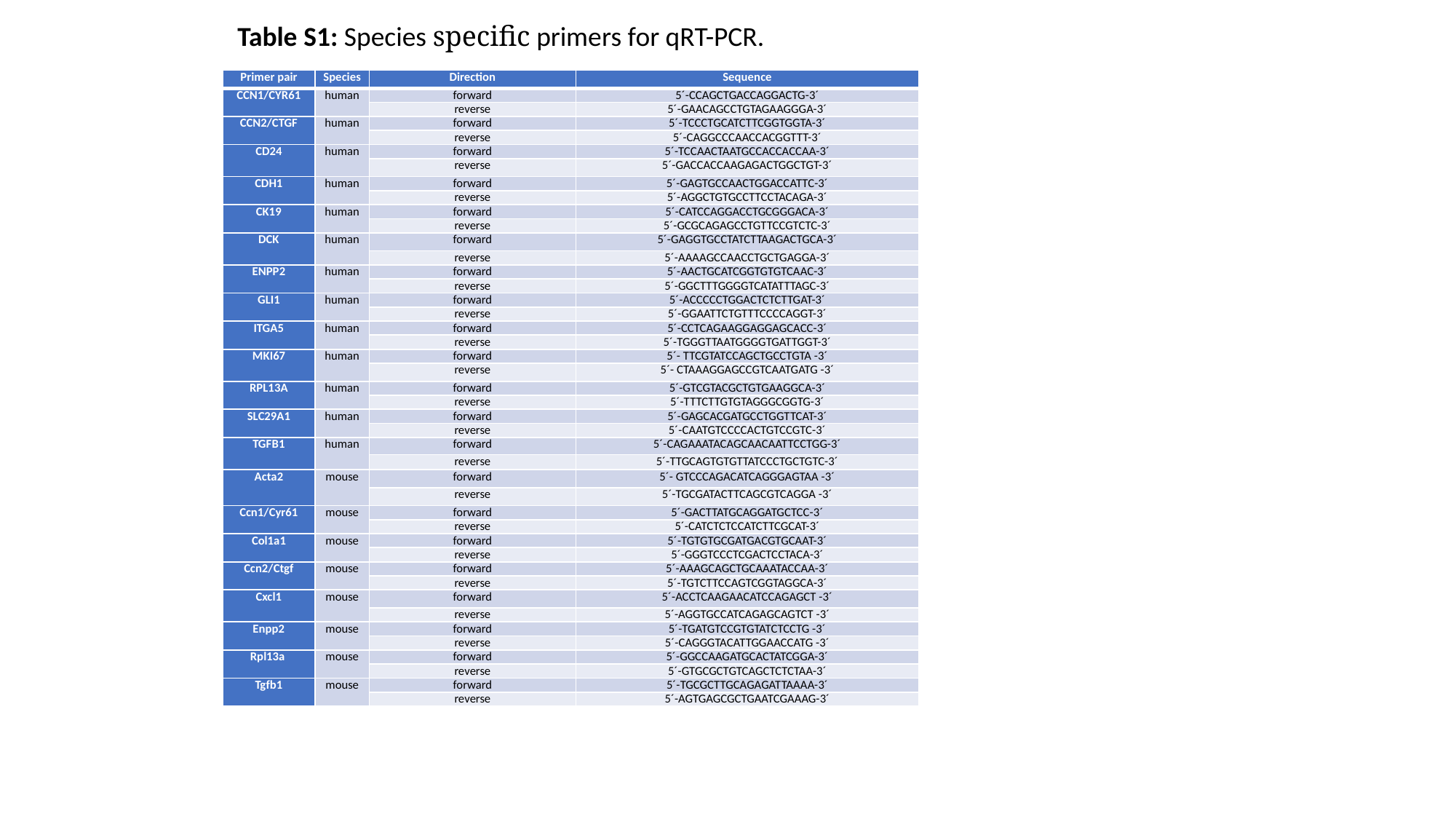

Table S1: Species specific primers for qRT-PCR.
| Primer pair | Species | Direction | Sequence |
| --- | --- | --- | --- |
| CCN1/CYR61 | human | forward | 5´-CCAGCTGACCAGGACTG-3´ |
| | | reverse | 5´-GAACAGCCTGTAGAAGGGA-3´ |
| CCN2/CTGF | human | forward | 5´-TCCCTGCATCTTCGGTGGTA-3´ |
| | | reverse | 5´-CAGGCCCAACCACGGTTT-3´ |
| CD24 | human | forward | 5´-TCCAACTAATGCCACCACCAA-3´ |
| | | reverse | 5´-GACCACCAAGAGACTGGCTGT-3´ |
| CDH1 | human | forward | 5´-GAGTGCCAACTGGACCATTC-3´ |
| | | reverse | 5´-AGGCTGTGCCTTCCTACAGA-3´ |
| CK19 | human | forward | 5´-CATCCAGGACCTGCGGGACA-3´ |
| | | reverse | 5´-GCGCAGAGCCTGTTCCGTCTC-3´ |
| DCK | human | forward | 5´-GAGGTGCCTATCTTAAGACTGCA-3´ |
| | | reverse | 5´-AAAAGCCAACCTGCTGAGGA-3´ |
| ENPP2 | human | forward | 5´-AACTGCATCGGTGTGTCAAC-3´ |
| | | reverse | 5´-GGCTTTGGGGTCATATTTAGC-3´ |
| GLI1 | human | forward | 5´-ACCCCCTGGACTCTCTTGAT-3´ |
| | | reverse | 5´-GGAATTCTGTTTCCCCAGGT-3´ |
| ITGA5 | human | forward | 5´-CCTCAGAAGGAGGAGCACC-3´ |
| | | reverse | 5´-TGGGTTAATGGGGTGATTGGT-3´ |
| MKI67 | human | forward | 5´- TTCGTATCCAGCTGCCTGTA -3´ |
| | | reverse | 5´- CTAAAGGAGCCGTCAATGATG -3´ |
| RPL13A | human | forward | 5´-GTCGTACGCTGTGAAGGCA-3´ |
| | | reverse | 5´-TTTCTTGTGTAGGGCGGTG-3´ |
| SLC29A1 | human | forward | 5´-GAGCACGATGCCTGGTTCAT-3´ |
| | | reverse | 5´-CAATGTCCCCACTGTCCGTC-3´ |
| TGFB1 | human | forward | 5´-CAGAAATACAGCAACAATTCCTGG-3´ |
| | | reverse | 5´-TTGCAGTGTGTTATCCCTGCTGTC-3´ |
| Acta2 | mouse | forward | 5´- GTCCCAGACATCAGGGAGTAA -3´ |
| | | reverse | 5´-TGCGATACTTCAGCGTCAGGA -3´ |
| Ccn1/Cyr61 | mouse | forward | 5´-GACTTATGCAGGATGCTCC-3´ |
| | | reverse | 5´-CATCTCTCCATCTTCGCAT-3´ |
| Col1a1 | mouse | forward | 5´-TGTGTGCGATGACGTGCAAT-3´ |
| | | reverse | 5´-GGGTCCCTCGACTCCTACA-3´ |
| Ccn2/Ctgf | mouse | forward | 5´-AAAGCAGCTGCAAATACCAA-3´ |
| | | reverse | 5´-TGTCTTCCAGTCGGTAGGCA-3´ |
| Cxcl1 | mouse | forward | 5´-ACCTCAAGAACATCCAGAGCT -3´ |
| | | reverse | 5´-AGGTGCCATCAGAGCAGTCT -3´ |
| Enpp2 | mouse | forward | 5´-TGATGTCCGTGTATCTCCTG -3´ |
| | | reverse | 5´-CAGGGTACATTGGAACCATG -3´ |
| Rpl13a | mouse | forward | 5´-GGCCAAGATGCACTATCGGA-3´ |
| | | reverse | 5´-GTGCGCTGTCAGCTCTCTAA-3´ |
| Tgfb1 | mouse | forward | 5´-TGCGCTTGCAGAGATTAAAA-3´ |
| | | reverse | 5´-AGTGAGCGCTGAATCGAAAG-3´ |
